# Crystal structure of the Cenp-HIK^Head^-TW sub-module of the inner kinetochore CCAN complex

**DOI:** 10.1093/nar/gkaa772

**Published:** 2020-09-25

**Authors:** Ziguo Zhang, Dom Bellini, David Barford

**Affiliations:** MRC Laboratory of Molecular Biology, Francis Crick Avenue, Cambridge CB2 0QH, UK; MRC Laboratory of Molecular Biology, Francis Crick Avenue, Cambridge CB2 0QH, UK; MRC Laboratory of Molecular Biology, Francis Crick Avenue, Cambridge CB2 0QH, UK

## Abstract

Kinetochores are large multi-subunit complexes that attach centromeric chromatin to microtubules of the mitotic spindle, enabling sister chromatid segregation in mitosis. The inner kinetochore constitutive centromere associated network (CCAN) complex assembles onto the centromere-specific Cenp-A nucleosome (Cenp-A^Nuc^), thereby coupling the centromere to the microtubule-binding outer kinetochore. CCAN is a conserved 14–16 subunit complex composed of discrete modules. Here, we determined the crystal structure of the *Saccharomyces cerevisiae* Cenp-HIK^Head^-TW sub-module, revealing how Cenp-HIK and Cenp-TW interact at the conserved Cenp-HIK^Head^–Cenp-TW interface. A major interface is formed by the C-terminal anti-parallel α-helices of the histone fold extension (HFE) of the Cenp-T histone fold domain (HFD) combining with α-helix H3 of Cenp-K to create a compact three α-helical bundle. We fitted the Cenp-HIK^Head^-TW sub-module to the previously determined cryo-EM map of the *S. cerevisiae* CCAN–Cenp-A^Nuc^ complex. This showed that the HEAT repeat domain of Cenp-I^Head^ and C-terminal HFD of Cenp-T of the Cenp-HIK^Head^-TW sub-module interact with the nucleosome DNA gyre at a site close to the Cenp-A^Nuc^ dyad axis. Our structure provides a framework for understanding how Cenp-T links centromeric Cenp-A^Nuc^ to the outer kinetochore through its HFD and N-terminal Ndc80-binding motif, respectively.

## INTRODUCTION

Accurate chromosome segregation in mitosis and meiosis underlies the successful inheritance of genetic information by future generations. In eukaryotes, duplicated sister chromatids pairs are aligned at the metaphase plate of the mitotic spindle until separated at the onset of anaphase. Chromosomes are physically connected to the mitotic spindle by kinetochores, large protein complexes that both assemble onto centromeric chromatin and attach to and track the plus end of microtubules (1,2). The physical movement of chromosomes is powered by microtubule depolymerization, pulling chromosomes to centrosomes at opposite poles of the cell. As well as acting as load-bearing elements, kinetochores control chromosome segregation fidelity by monitoring tension and microtubule attachment to ensure biorientation, integrating this information to activate error correction mechanisms and the spindle assembly checkpoint (3,4).

Kinetochores, comprising over 50 different proteins, are delineated into the inner and outer kinetochore. The inner kinetochore specifically assembles onto the centromere-specific Cenp-A nucleosome (Cenp-A^Nuc^) through the CCAN complex. CCAN then links the centromere to the KMN network that forms the outer kinetochore. The Ndc80 complex (Ndc80c) of the KMN network, together with the DASH/Dam1 complex of *Saccharomyces cerevisiae* and the Ska complex of vertebrates, attach to microtubules (5). The error-correction, tension sensing chromosome passenger complex (CPC) is located at the inner kinetochore by interacting with both CCAN and a proximal H3-nucleosome, whereas proteins responsible for the spindle assembly checkpoint (SAC) assemble at the outer kinetochore through the KNL1 complex of the KMN network.

The 16 components of the vertebrate CCAN (6–9) share structural similarities to proteins of the 14-subunit budding yeast Ctf19 complex discovered earlier (10,11). Cenp-T, comprising a C-terminal histone fold domain (HFD) co-purifies and interacts with a novel HFD protein Cenp-W as a DNA-binding heterodimer (12–14). In vertebrates, Cenp-TW together with two other HFD proteins, Cenp-S and Cenp-X, generate a Cenp-TWSX heterotetramer (15). The structural resemblance of Cenp-TWSX to the histone H3-H4 heterotetramer, and its ability to bind and supercoil DNA, suggested the possibility that Cenp-TWSX forms a nucleosome-like structure that contacts centromeric DNA (15). Cenp-TW interacts directly with Cenp-HIKM to form a six-subunit complex (Cenp-HIKM-TW) in vertebrates (16,17), and a five-subunit complex (Cenp-HIK-TW) in budding yeast (13,14).

It is now clear that most of the centromere proteins (Cenps) constituting the CCAN form discrete sub-complexes; Cenp-OPQU, Cenp-LN and Cenp-HIK-TW (14,16–20). Cenp-C and Cenp-N specifically recognize Cenp-A^Nuc^ (21–24), with Cenp-C also interacting with Cenp-LN (16,17,25), Cenp-HIKTW (16,17) (and our unpublished data for budding yeast), and in budding yeast, Cenp-OPQU+ (26). Cenp-C directly links Cenp-A^Nuc^ to the outer kinetochore through its interactions with the Mis12 complex (Mis12c) of the KMN network, an evolutionarily conserved interaction network (27–29). The budding yeast Cenp-U of the Cenp-OPQU+ complex also interacts with Mis12c (26,29), with recent data showing that Cenp-OPQU+, through Cenp-Q, interacts with the N-terminus of the Cenp-A histone (30,31). Finally, the intrinsically disordered N-terminus of Cenp-T connects to the outer kinetochore through Ndc80c (13,32,33). Vertebrate Cenp-T forms additional interactions with Mis12c (34), providing another link to Ndc80c and microtubules.

Recently cryo-EM studies of the *S. cerevisiae* CCAN complex (Ctf19 complex) revealed the overall atomic-resolution structure of the complex (35–37). Cenp-OPQU, which forms a six-subunit subcomplex with Nkp1 and Nkp2 (termed Cenp-OPQU+) (20), forms an extensive and stable interface with Cenp-LN to define a globular core. Cenp-I of Cenp-HIK interacts with Cenp-L through a small contact surface that allows conformational flexibility of the Cenp-HIK module. In both published structures, only the main body of Cenp-HIK (Cenp-HIK^Body^) was visualized to atomic resolution. In Cenp-HIK^Body^, the C-terminal HEAT domain of Cenp-I runs anti-parallel to the coiled-coil α-helices of Cenp-H and Cenp-K. Because of conformational variability of Cenp-HIK^Head^ (N-terminal HEAT domain of Cenp-I and C-terminal segments of Cenp-H and Cenp-K) cryo-EM density for this sub-module was not clearly defined, although density matching the crystal structure of thermophilic yeast Cenp-HIK^Head^ (38) was visible, allowing an approximate placing of Cenp-HIK^Head^ in the overall cryo-EM maps. In the CCAN – Cenp-A^Nuc^ complex, Cenp-HIK^Head^ interacts with the DNA gyre of Cenp-A^Nuc^. However, the DNA-binding HFDs of Cenp-TW, that interact with Cenp-HIK^Head^ (37), could not be built into the cryo-EM density maps, and thus their mode of interaction with DNA could not be defined. In this paper we have determined the crystal structure of *S. cerevisiae* Cenp-HIK^Head^-TW, which we then fitted into the published cryo-EM CCAN–Cenp-A^Nuc^ structure. This indicates that Cenp-TW is positioned on Cenp-HIK^Head^ to interact with the DNA gyre of Cenp-A^Nuc^ at a site close to the Cenp-A^Nuc^ dyad axis, opposite to the Cenp-LN DNA-binding channel.

## MATERIALS AND METHODS

### Cloning, expression, purification and crystallization of Cenp-HIK^Head^-TW

Coding fragments of *CTF3^1^^–^^245^* (*CENP-I^N^*), *MCM16^137^^–^^182^* (*CENP-H^C^*), *MCM22^130^^–^^239^* (*CENP-K^C^*), and full-length *CNN1* (*CENP-T*) and *WIP1* (*CENP-W*) previously amplified by PCR from *S. cerevisiae* genomic DNA (37) were cloned into the pU1 baculovirus expression vector (39). The gene expression cassettes for *CTF3^1^^–^^245^, MCM16^137^^–^^182^, MCM22^130^^–^^239^, CNN1* and *WIP1* were further cloned into pF2 for generating a virus to express the Cenp-HIK^Head^-TW complex (Head: denotes truncated Cenp-H, Cenp-I and Cenp-K proteins representing the Cenp-HIK^Head^ domain as defined by (37)) using a modified MultiBac expression system (39). A double StrepII tag together with a TEV cleavage site were attached to the C-terminus of the Ctf3^1-245^ protein. The same coding regions for the Cenp-HIK^Head^-TW complex were cloned into the pET28 plasmid for selenomethionine labelling using an *E. coli* expression system. For Cenp-TW expression, full length *MCM16, MCM22, CNN1* and *WIP1* were cloned into the MultiBac expression system, with a double StrepII tag attached to the C-terminus of Cenp-T.

The Cenp-HIK complex was recombined by co-expressing full length *CTF3* with *MCM16 (CENP-H)* and *MCM22 (CENP-K)*. The constructs for reconstituting complexes with mutations of Cenp-HI^T91Y^K, Cenp-T ^L350R/S354/Y/H345R^ W, Cenp-HI ^R215A/K225A^ KT ^L350R/S354/Y/H345R^W were generated by USER methodology using full-length proteins (39).

All complexes except selenomethionine labeled Cenp-HIK^Head^-TW were expressed using the baculovirus-insect cell system as described (39). The cell pellet was lysed in a buffer of 50 mM Tris–HCl (pH 8.0), 200 mM NaCl, 1 mM DTT, 1 mM EDTA and loaded onto a Strep-Tactin column, and eluted in 5 mM desthiobiotin and the NaCl diluted to 100 mM for loading onto a Resource Q anion exchange column. The protein was then purified by size exclusion chromatography on a Superdex 200 size exclusion chromatography in buffer of 20 mM HEPES (pH 8.0), 150 mM NaCl, 1 mM DTT, 1 mM EDTA. Selenomethionine labeled Cenp-HIK^Head^-TW complex was produced in *Escherichia coli* using SeMet Medium Base Plus Nutrient Mix and Seleno Methionine Solution (Molecular Dimensions Ltd.). In brief, the cells were grown at 37°C, shaken at 220 rpm to an OD_600_ of 0.6. Protein expression was induced by addition of 0.3 mM IPTG, and the culture was incubated at 20°C, 220 rpm for 16 h. The complex purification was performed as for the baculovirus/insect cell expression of native protein, except that 10 mM DTT and 5 mM EDTA were used in the buffers. For crystallization, the protein was concentrated to 5 mg/ml in a buffer containing 20 mM HEPES (pH 8.0), 150 mM NaCl, 1 mM EDTA and 1 mM DTT. Initial crystals were obtained by vapour diffusion in sitting drops in condition H11 of LMB 21 screening plate (sparse matrix screen, MD1-98) (40), containing 1.6 M NaK_2_PO_4_. The crystals were optimized in hanging drops with 1 M NaH_2_PO_4_ and 0.38 M K_2_HPO_4_. Selenomethionine labelled Cenp-HIK^Head^-TW crystals grew in similar conditions with 10 mM DTT. Crystals were incubated in a cryoprotection buffer comprising 1 M NaH_2_PO_4_ and 0.38 M K_2_HPO_4_ and 25% glycerol prior to freezing in liquid nitrogen.

### Crystallographic data collection and reduction

The high-resolution native dataset was collected at 100 K on beamline I24 at Diamond Light Source (DLS), Didcot, UK at a wavelength within the lead L-III absorption edge of 0.9465 Å using a PILATUS3 6M detector (DECTRIS) with a crystal-to-detector distance to allow diffraction to 3 Å resolution at the detector edge. The eight collections were auto-processed using the XDS pipeline (41) in Xia2 (42) and merged together into a single dataset with Aimless (43). For the Se-SAD experiment, single datasets from 31 randomly orientated crystals were recorded at 100 K on beamline I03 at a wavelength of 0.9793 Å using an Eiger2 XE 16M (DECTRIS) with a crystal-to-detector distance to cover diffraction to 2.9 Å resolution at the detector edge. The optimal cluster of isomorphous datasets was obtained using the program BLEND (44). Selected datasets were auto-processed using the DIALS pipeline (45) in Xia2 and merged together into a single dataset with Aimless (43). Native data used in structure determination and refinement were anisotropically corrected using the STARANISO server (Tickle, I.J., Flensburg, C., Keller, P., Paciorek, W., Sharff, A., Vonrhein, C., Bricogne, G. (2018). STARANISO (http://staraniso.globalphasing.org/cgi-bin/staraniso.cgi). Cambridge, United Kingdom: Global Phasing Ltd.), however, isotropically processed intensities labelled IMEAN_iso (and SIGIMEAN_iso) have also been deposited to the Protein Data Bank.

### Structure determination, refinement and validation

The high-resolution dataset of the Cenp-HIK^Head^-TW complex referred to as ‘native’ in this study was collected at the lead L-III absorption edge from a crystal that had been soaked with 20 mM trimethyl lead acetate (TMLA) for 72 h prior to cryo-freezing. The resulting data set extended anisotropically to 2.9 Å (Table 1). Pb-SAD phasing attempts failed despite detection of weak anomalous signal during data reduction. Molecular replacement using the Cenp-HIK^Head^ heterotrimer (PDB: 5Z08) (37) and Cenp-TW heterodimer (PDB: 3B0C) (15) as search models, also failed to give correct solutions.

**Table 1.** Table of crystallographic data collection and refinement statistics

Cenp-HIK^Head^-TW (PDB: 6YPC)	Se-Met	Native
**Data collection**		
Beamline	I03, DLS	I24, DLS
Wavelength	0.9793	0.9465
Resolution range (Å)^a^	3.8–69 (3.8–4)	2.9–74 (2.9–3.07)
Space group	*I*4_1_22	*I*4_1_22
Unit cell parameters (Å)	*a* = *b* = 133.1, *c* = 242	*a* = *b* = 132.58, *c* = 241.68
No. of crystals	11	1 (8-wedge series)
No. of unique reflections	11126	24344
Multiplicity^a^	316 (335)	63.3 (62.3)
Completeness (%)^a^	100 (99.6)	100 (100)
Mean I/sigI^a^	13 (1.6)	10 (1.1)
*R*_meas_^b^	0.41 (13.8)	0.37 (14.5)
*R*_pim_	0.024 (0.75)	0.047 (1.83)
CC_1/2_^a^	1.0 (0.81)	1.0 (0.68)
**Refinement**		
Resolution (Å)		69–2.9
*R*_work_^c^		0.21
*R*_free_^d^		0.26
R.m.s.d., bonds lengths (Å)		0.01
R.m.s.d., angles (°)		1.3
Mean B factor (Å^2^)		85
Wilson B factor (Å^2^)		101
Ramachandran favoured/allowed (%)		90/8
Rotamer outliers		0
Molprobity score		2.43
RCSB PDB ID	-	6YPC

^a^Parentheses indicate the highest-resolution shell.

^b^ *R*_meas_ = {∑_*hkl*_√*n*/(*n*− 1)[∑^*n*^_*j* = 1_|*I_hkl,j_*− 〈*I_hkl_*&x3009 x232A;|]}/∑_*hkl*_∑*_j_I_hkl,j_*.

^c^ *R*_work_ = ∑||*F*_obs_| − |*F*_calc_||/|*F*_obs_| × 100.

^d^ *R*_free_, based on 5% of the total reflections.

Se-SAD experiments were therefore pursued by merging together only the first 200° wedge from a cluster of eleven isomorphous datasets out of 31 collections from randomly orientated SeMet crystals to obtain a dataset that extended to 3.8 Å (Table 1), and that showed significant anomalous signal up to 6 Å. An overall signal-to-noise ratio of 13 was deemed sufficient to phase a pentameric complex of 860 residues containing 10 selenomethionines resulting in a Bijvoet ratio of 4.2%. The selenium substructure was determined using the HKL2MAP graphical interface (46) with SHELXC, SHELXD and SHELXE (47). Multiple searches for the correct selenium substructure were performed over the 3.2–8 Å resolution range, estimating one heteropentamer in the asymmetric unit using the program MATTHEWS_COEF (48). A solution was obtained at 6.5 Å showing at least five heavy atom sites. Successful phasing was achieved with phenix.autosol (49) generating maps at 6.5 Å with clear α-helical features. The search models 5Z08 and 3B0C for heterotrimeric Cenp-HIK, and dimeric Cenp-TW, respectively, succeeded in a MOLREP (50) run using the phased translation function. This newly assembled heteropentamer model was successfully utilized in a MOLREP run against the high-resolution native structure factors, providing a solution that could be refined initially with REFMAC5 (51) and at later stages with phenix.refine (49). Manual building was carried out with Coot (52). Crystallographic statistics are listed in Table 1. The difference anomalous maps calculated for the dataset from the TMLA-soaked crystal using the phases of the refined structure failed to localize any lead site confirming that these data are indeed native. An example of a 2Fo-Fc density map is shown in Supplementary Figure S1A, B. MolProbity (53) was used for model validation.

Structural conservation was analysed using Consurf (54,55). The Cenp-HIK^Head^-TW crystal structure was fit to the cryo-EM density maps of CCAN – Cenp-A^Nuc^ and apo dimeric CCAN (37) using Chimera (56). This was possible because a 3D cryo-EM class of the CCAN–Cenp-A complex (EMD-11626) revealed EM density for the Cenp-HIK^Head^-TW sub-module. The remaining subunits of CCAN are as reported previously (37), except that we used the recent cryo-EM model of *S. cerevisiae* Ctf3c–Cnn1–Wip1 (Cenp-HIK-TW) (PDB 6WUC) (57) to fit the ‘flexible ‘joint’ region that connects Cenp-HIK^Body^ to CenpHIK^Head^: residues 139–142 of Cenp-H, residues 285–353 of Cenp-I, and residues 141–148 of Cenp-K as a rigid body fit in Coot (52), and additionally, the α-helix (residues 242–268) to Cenp-I^Head^. The fit of Cenp-HIK^Head^-TW into the CCAN – Cenp-A^Nuc^ cryo-EM map (EMD-11626) was assessed using the Chimera-derived correlation coefficient between the calculated EM density of the fitted models and the cryo-EM map. The correlation coefficient for the fit of Cenp-HIK^Head^-TW to the assigned density is 0.926, slightly lower than that for the fit of Cenp-HIK^Body^ (0.953). The higher correlation coefficient for Cenp-HIK^Head^ alone (0.955), likely results from the weaker density for Cenp-TW compared with Cenp-HIK^Head^. The correlation coefficient for randomly placing Cenp-HIK^Head^-TW into Cenp-HIK^Body^ density was 0.880, clearly distinct from the fits into the assigned HIK^Head^-TW density. Figures were generated using PyMOL (Molecular Graphics Systems, 2.03, Schrodinger) and Consurf (54,55).

### Cenp-HIK–Cenp-TW interaction analysis

To test the Cenp-HIK–Cenp-TW interaction and consequence of disrupting this interface on their mutual interaction, wild type and mutant forms of Cenp-HIK and Cenp-TW were mixed and incubated at equal molar ratios of 10 μM at 4°C for 1 h in a buffer of 10 mM Tris–HCl (pH 8.0), 150 mM NaCl, 1 mM EDTA, 1 mM DTT, and then resolved on a Micro-S200 size exclusion chromatography column.

## RESULTS AND DISCUSSION

### Cenp-HIK^Head^-TW architecture

**Figure 1.**
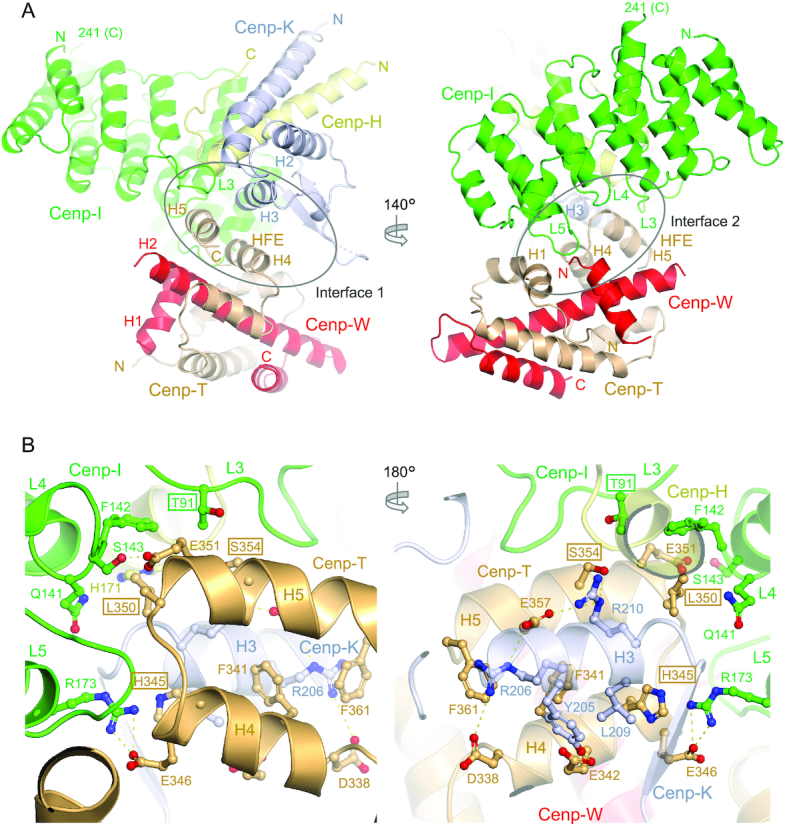
Overall view of the Cenp-HIK^Head^-TW complex. (**A**) Two views of the complex with interface 1 shown on the left and interface 2 on the right. (**B**) Two views of the Cenp-HIK^Head^-TW interface 1. The interface is dominated by the HFE of Cenp-T forming a three α-helical bundle with H3 of Cenp-K. Residues mutated in this study are boxed.

The structure of the *S. cerevisiae* Cenp-HIK^Head^ sub-module at 2.9 Å resolution is essentially identical to its counterpart in the thermophilic yeast (38) (Figure 1A and Supplementary Figure S2A). Cenp-HIK^Head^ is assembled from the N-terminus of Cenp-I^Head^ (residues 1–241) and the C-termini of Cenp-H (residues 147–181, Cenp-H^Head^) and Cenp-K (residues 136–237, Cenp-K^Head^). Cenp-I^Head^ is composed of five HEAT repeat-like motifs that generate a double layer of α-helices, capped at its C-terminus by a single α-helix, which then connects to a linker region running along the Cenp-I^Head^ HEAT-domain to form a 13th α-helix located at the N-terminus of the domain (Figure 1A). The single α-helix of Cenp-H^Head^ inserts between Cenp-I^Head^ and Cenp-K^Head^. Cenp-K^Head^ comprises three α-helices followed by a short two-stranded β-sheet. Three loops that connect the pair of α-helices of three contiguous HEAT repeats (L3, L4 and L5), contribute to the interface of Cenp-HIK^Head^ with Cenp-TW (Figure 1A).

*S. cerevisiae* Cenp-TW resembles that of chicken Cenp-TW (15) (Figure 1A and Supplementary Figure S2B), which are in turn related structurally to the canonical histone dimers of H2A-H2B and H3-H4. Differing from chicken Cenp-W, a disordered 20-residue loop connects α-helices H1 and H2 in *S. cerevisiae* Cenp-W. Cenp-W comprises a canonical histone fold domain (HFD) of three α-helices, whereas Cenp-T includes a HFD followed by two solvent exposed α-helices (H4 and H5) termed the histone fold extension (HFE) (13–15). Although we crystallized a complex of Cenp-HIK^Head^-Cenp-TW using the full-length Cenp-T subunit, in our structure the N-terminal 268 residues are disordered, consistent with disorder predictions, with only the HFD and HFE being visible.

The crystal structure of the Cenp-HIK^Head^-TW sub-module reveals that the major contacts between Cenp-HIK^Head^ and Cenp-TW involve Cenp-I^Head^ and Cenp-K^Head^ of Cenp-HIK and Cenp-T of Cenp-TW, with Cenp-H^Head^ and Cenp-W making minor contributions (Figure 1A). Consistent with our structure, it was previously shown that the interaction of Cenp-T with Cenp-HIK^Head^ is not dependent on Cenp-W (14). Together, interactions between Cenp-TW and Cenp-HIK^Head^ comprise two contiguous interfaces. In interface 1, the C-terminal anti-parallel α-helices (H4 and H5) of the HFE of Cenp-T combine with H3 of Cenp-K to create a compact three helix bundle (Figure 1A). This interface is augmented by the Cenp-I^Head^ HEAT repeat domain that contributes a long loop (L3, residues 89–104). This loop in turn is stabilized through contacts with Cenp-H and Cenp-K (Thr91, Val94 and Arg97 of Cenp-I^Head^). Interface 1 is dominated by a network of electrostatic interactions involving Arg206 and Arg210 of Cenp-K and Asp338, Glu357 and Ser354 of Cenp-T (Figure 1B). Phe341 of Cenp-T buttresses the Cenp-K Arg206 and Arg210 side-chains. Other electrostatic interactions include Glu342 of Cenp-T with Tyr205 of Cenp-K (Figure 1B).

**Figure 2.**
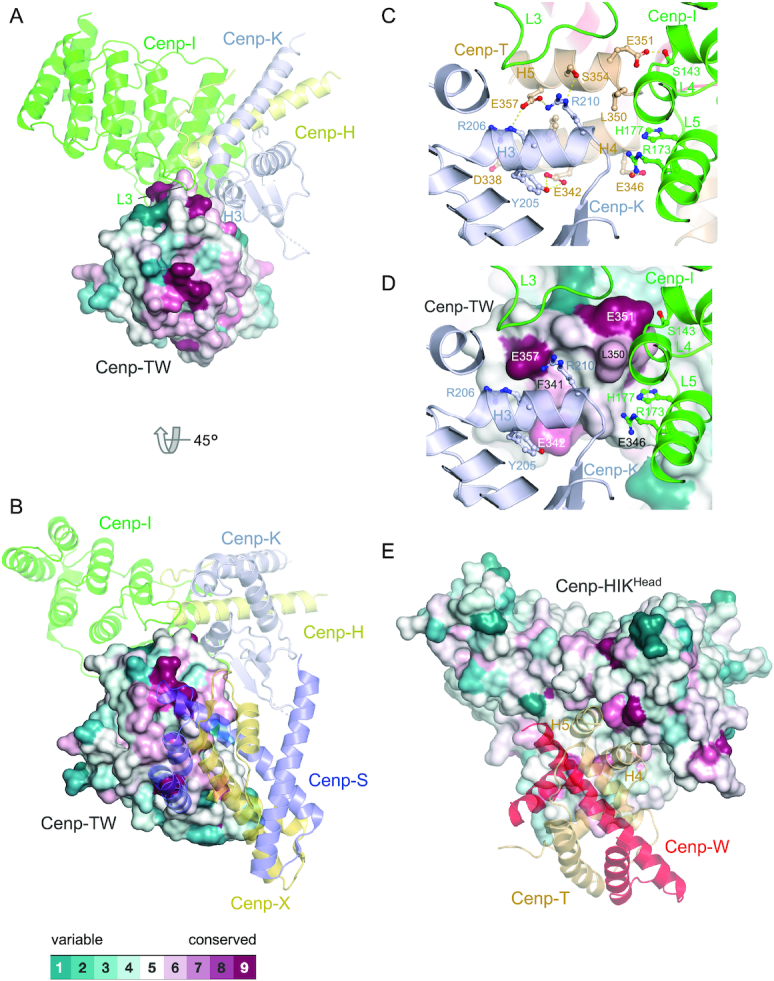
The Cenp-HIK^Head^-TW interface is conserved. (**A**) Structural conservation mapped onto the surface of Cenp-TW with Cenp-HIK^Head^ shown as a cartoon representation. (**B**) View in (A) rotated by 45° and with Cenp-SX modelled according to chicken Cenp-TWSX [PDB: 3VH5] (15). (C) and (D) Close-up views of the Cenp–HIK^Head^–TW interface 1, shown in cartoon representation in (**C**) and with structural conservation mapped onto Cenp-TW in (**D**). This shows the strong conservation of acidic residues Cenp-T^E342^, Cenp-T^E351^ and Cenp-T^E357^, and also Cenp-T^L350^ at this interface. (**E**) Structural conservation mapped onto the surface of Cenp-HIK^Head^ with Cenp-TW shown as a cartoon representation. Sequence conservation was based on the sequence alignment shown in Supplementary Figure S4. Conservation was determined using Consurf (54,55).

In interface 2, the conserved L5 loop (residues 177–182) of Cenp-I^Head^ inserts into a cavity within the Cenp-TW dimer formed by the interface of H1 of the Cenp-T HFD, turn connecting H4-H5 of the Cenp-T HFE, and N-terminus of Cenp-W (Figure 1A). His177^Cenp-I^ in the L5 loop, a highly conserved residue, makes an important contribution by providing a C-terminal helix cap to H4 of Cenp-T (Figure 2C, D and Supplementary Figure S3). The nearby Arg173 of Cenp-I forms an electrostatic interaction with Glu346 of Cenp-T. Leu350 of the Cenp-T H4–H5 turn bridges interfaces 1 and 2, forming non-polar contacts with all three chains of Cenp-HIK, specifically Phe142 of Cenp-I (Figure 1B). Additionally, the Cenp-I L4 loop contacts the H4-H5 turn of Cenp-T in which the highly conserved Glu351 accepts a hydrogen bond from Ser143 of Cenp-I (Figure 1B).

**Figure 3.**
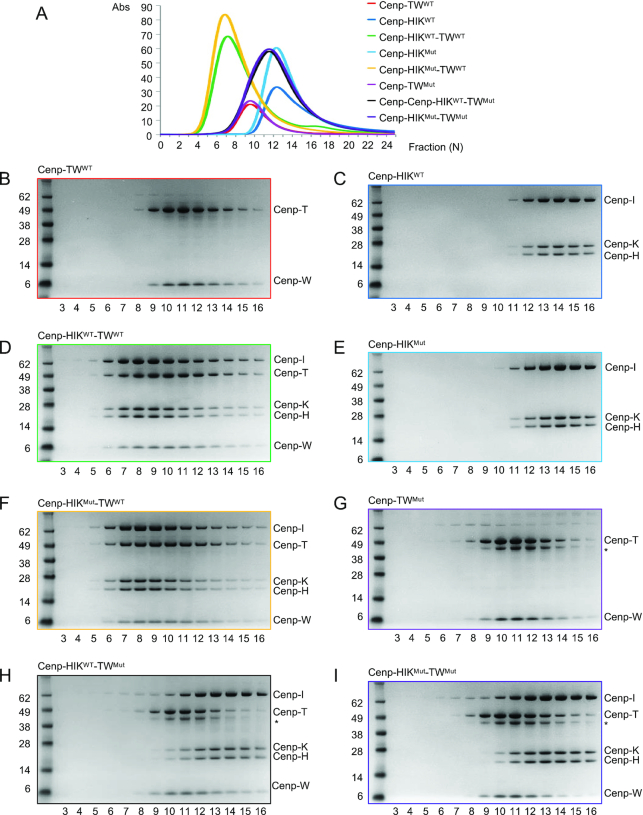
Mutating Cenp-T disrupts Cenp-HIK^Head^ – Cenp-TW interactions. (**A**) Size exclusion chromatograms of wild type (WT) and mutant Cenp-HIK and Cenp-TW sub-complexes. (**B–I**) Corresponding SDS PAGE gels of peak fractions from size exclusion columns. Cenp-HIK^Mut^ (Cenp-I: T91Y), Cenp-TW^Mut^ (Cenp-T: L350R/S354Y/H345R). * A co-purifying contaminant from Cenp-TW^Mut^.

The HFE of Cenp-T (Cenp-T^HFE^) was previously implicated in mediating Cenp-TW interactions with Cenp-HIK from *in vitro* reconstitution studies (14). In this study, mutating the three residues of Cenp-T^HFE^ (Glu346, Leu350, Glu351), that our structure shows mediates contacts at the Cenp-TW – Cenp-HIK^Head^ interface (Figure 1B), revealed that Cenp-TW failed to associate with Cenp-HIK, whereas contacts with Cenp-W were retained (14). To further assess our structure, we mutated His345, Leu350 and Ser354 (L350R, S354Y and H345R) of Cenp-T and Thr91 of Cenp-I (T91Y) (full-length proteins). As assessed by size exclusion chromatography (SEC), Cenp-HIK^Mut^ and Cenp-TW^Mut^ assembled correctly. Mutating Cenp-T alone, but not Cenp-I alone, abrogated the Cenp-HIK and Cenp-TW association (Figure 3). We assume that in the Cenp-I T91Y mutant, conformational changes of the solvent exposed Tyr91 side chain compensate for its substitution for Thr91.

### Structural conservation

Residues at the Cenp-HIK^Head^–Cenp-TW interface are highly conserved across eukaryotes, as shown by multiple sequence alignments of the regions of Cenp-I, Cenp-K and Cenp-T responsible for mediating the Cenp-HIK^Head^ – Cenp-TW interface (Supplementary Figure S4). Sequence conservation is mapped onto the surfaces of Cenp-HIK^Head^ and Cenp-TW in Figure 2. The electrostatic interactions at the interface involving Arg206 and Arg210 of Cenp-K, His177 of Cenp-I with Glu342, Glu351 and Glu357 of Cenp-T^HFE^, are well conserved from yeast to vertebrates. Notably Glu351 of Cenp-T^HFE^ is an invariant residue, and the neighbouring Leu350, located at the hub of the Cenp-T interface with Cenp-I and Cenp-K, is also highly conserved, and as mentioned earlier, mutation of Leu350 and Glu351 ablates Cenp-HIK – Cenp-TW interactions (this work and (14)). This supports the view that the Cenp–HIK^Head^–Cenp-TW architecture will be evolutionarily conserved.

### Fitting Cenp-HIK^Head^-TW to CCAN–Cenp-A cryo-EM structure

**Figure 4.**
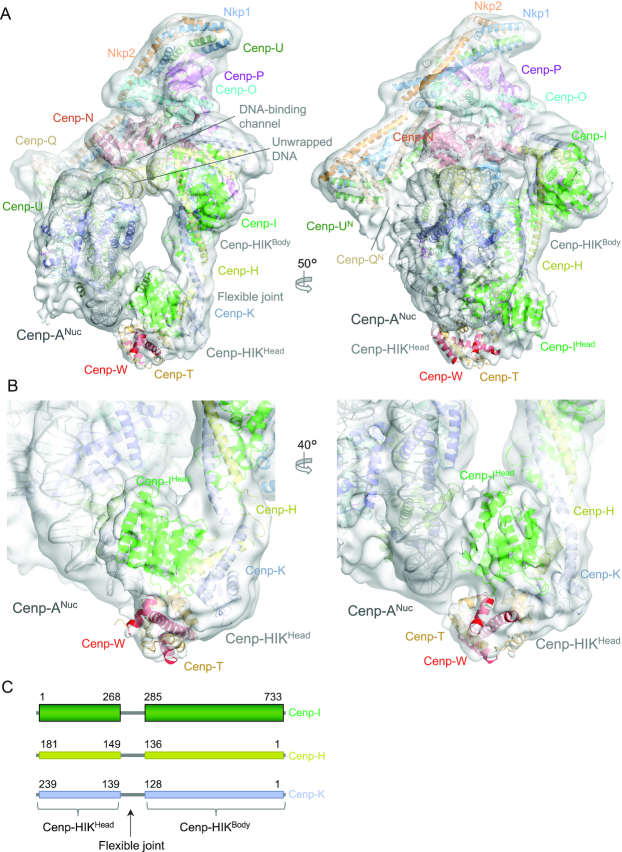
The Cenp-HIK^Head^-TW sub-module contacts the DNA gyre of Cenp-A^Nuc^. (**A**) Two views of the cryo-EM density map (transparent surface) of the CCAN – Cenp-A^Nuc^ complex (37) with the atomic model shown as a cartoon representation. This shows the fit of the Cenp-HIK^Head^-TW crystal structure into EM density associated with Cenp-HIK^Body^ and adjacent to DNA of Cenp-A^Nuc^. (**B**) Two close-up views of the cryo-EM density of the CCAN–Cenp-A^Nuc^ complex with fitted Cenp-HIK^Head^-TW. On the right CenpI^Head^ and Cenp-T are shown in contact with the DNA gyre of Cenp-A^Nuc^. (**C**) Schematic of the Cenp-HIK module defining Cenp-HIK^Head^ and Cenp-HIK^Body^.

**Figure 5.**
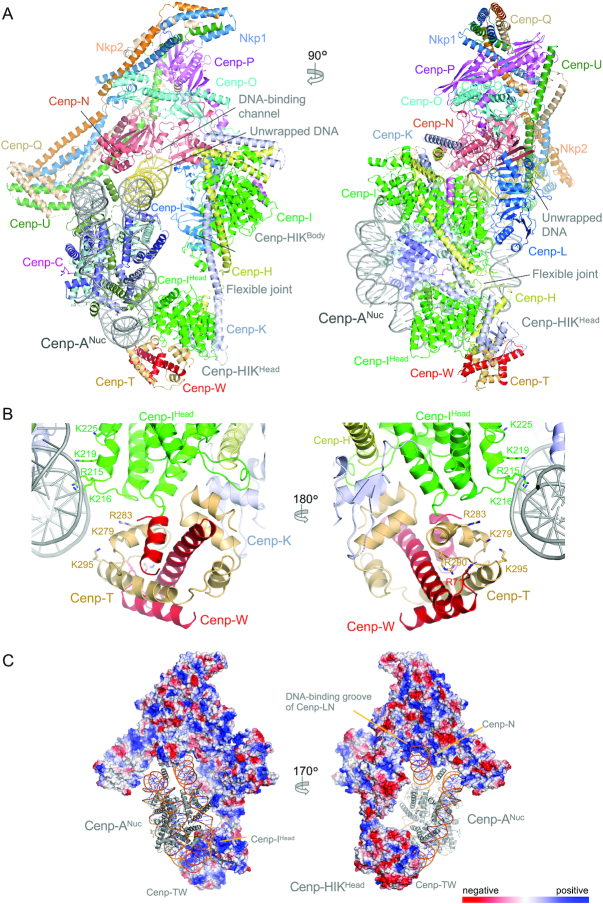
Model of CCAN – Cenp-A^Nuc^ with fitted Cenp-HIK^Head^-TW. (**A**) Two orthogonal views of the CCAN – Cenp-A^Nuc^ complex with the fitted Cenp-HIK^Head^-TW sub-module. (**B**) Two close-up views of the fitted Cenp-HIK^Head^-TW sub-module showing basic residues of Cenp-I^Head^ and Cenp-T that interact with the DNA gyre of Cenp-A^Nuc^. Of the residues indicated, Cenp-I^K216^, Cenp-T^K279^, Cenp-T^K295^, Cenp-W^R71^ are conserved in *H. sapiens*. (**C**) Regions of CCAN that contact the Cenp-A^Nuc^ DNA duplex feature positive electrostatic surfaces. The left panel shows the positively charged surface on Cenp-HIK^Head^-TW that contacts the DNA gyre close to SHL3. Right panel shows the positively charged DNA-binding groove of the Cenp-LN channel that engages the unwrapped DNA duplex at the terminus of Cenp-A^Nuc^.

We fitted the Cenp-HIK^Head^-TW domain into the cryo-EM density of the CCAN–Cenp-A complex (37) (Figure 4). This was possible because a 3D class of the CCAN – Cenp-A complex (EMD-11626) revealed EM density for Cenp-HIK^Head^-TW. This showed a good fit of the Cenp-HIK^Head^-TW crystal structure with the assigned density connected to the body of Cenp-HIK (Figure 4). The Cenp-HIK^Head^-TW sub-module contacts the DNA gyre of the Cenp-A nucleosome close to the dyad axis and SHL3, opposite the DNA-binding channel of Cenp-LN that engages the unwrapped DNA duplex at one of the Cenp-A^Nuc^ DNA termini (Figures 4A and 5A) (37). Plotting the electrostatic potential of CCAN with the fitted Cenp-HIK^Head^-TW sub-module (Figure 5C) reveals that the regions of CCAN that contact the DNA gyre of Cenp-A^Nuc^ are positively charged. These include the Cenp-LN-DNA binding channel (37) and a positively-charged surface generated by the combination of Cenp-I^Head^ and Cenp-T that is directed toward the Cenp-A DNA gyre. However, the exact basic residues that create this positively-charged surface are not well conserved in Cenp-I and Cenp-T homologs (Figure 5B). We were unable to isolate stable Cenp–HIK^Head^–TW complexes with Cenp-A^Nuc^, preventing us from testing the functional roles of the basic residues of Cenp-I^Head^ and Cenp-T that contact the DNA gyre.

Cenp-A nucleosomes with a right-handed DNA configuration have been proposed (58), although in crystal and cryo-EM structures of yeast and vertebrate Cenp-A^Nuc^, the DNA is left-handed. We explored whether a Cenp-A nucleosome with a right-handed DNA chirality would be compatible with CCAN assembly. We assumed that a right-handed Cenp-A^Nuc^ is an octasome with similar dimensions to the left-handed Cenp-A^Nuc^. Aligning the unwrapped DNA of the left-handed Cenp-A^Nuc^ (defined by its interaction with the Cenp-LN DNA-binding groove) onto the equivalent segment of the modelled right-handed Cenp-A^Nuc^, indicates that the DNA gyre wrapping the histone octamer, located between the unwrapped DNA and Cenp-QU in the left-handed Cenp-A^Nuc^ (Figure 5A, left panel), would instead be positioned between the unwrapped DNA segment and Cenp-HIK for a right-handed Cenp-A^Nuc^. In this situation the DNA gyre would clash with Cenp-HIK^Body^, and less severely with Cenp-HIK^Head^ (data not shown). However, it is possible that these steric clashes may be alleviated by rotating Cenp-A^Nuc^, and/or conformational changes of CCAN. Thus, this analysis suggests that the proposed right-handed Cenp-A^Nuc^ might be less suited to bind CCAN.

### Comparison of yeast Cenp-HIK^Head^-TW and chicken Cenp-TWSX

In vertebrates, Cenp-TW associates with the histone fold-like proteins Cenp-S and Cenp-X to form a Cenp-TWSX heterotetramer (15), reminiscent in architecture to the H3-H4 tetramer. Cenp-TWSX interacts with and supercoils DNA and has been proposed to form a nucleosome-like particle to contribute to kinetochore attachment to chromatin (12,15). In budding yeast, ChiP-seq studies indicated that binding of Cenp-TW occurs at the core of the centromere (14), and that Cenp-TW does not form a separate nucleosome-like particle. This result is consistent with our cryo-EM structure of the CCAN–Cenp-A complex showing that a modelled Cenp-HIK^Head^-TW sub-module would contact the DNA gyre of the Cenp-A nucleosome (37). Budding yeast Cenp-TW closely resembles chicken Cenp-TW (15) (Supplementary Figure S2B). A notable difference however, anticipated in an earlier study (13), is that the central α-helix H2 of the Cenp-T HFD is three turns longer in chicken Cenp-T than in *S. cerevisiae*. In the chicken Cenp-TWSX complex, this region of Cenp-T interacts with Cenp-S. We and others were unable to isolate a complex of Cenp-TW with the Cenp-SX homologs Mhf1 and Mhf2 using recombinant proteins (14), and Mhf1/Mhf2 did not associate with a kinetochore-Cenp-A nucleosome complex assembled *de novo* using yeast extracts (59). However, Mhf1 was previously reported to co-purify with kinetochore subunits in yeast extracts (14). Our structural study showing that the site on chicken Cenp-TW that binds to Cenp-SX is conserved in *S. cerevisiae* Cenp-TW (Figure 2B), leaves open the possibility that the budding yeast Cenp-SX homologs interact with the yeast kinetochore in the context of centromeric chromatin. Furthermore, in the chicken Cenp-TWSX complex, Cenp-SX binds to a site on Cenp-TW that is opposite to the Cenp-HIK^Head^-binding site of budding yeast Cenp-TW, thus compatible with Cenp-HIK^Head^–Cenp-TW interactions (Figure 2B).

### Conformational flexibility of the Cenp-HIK^Head^-TW sub-module

We analysed the conformational variability of the Cenp-HIK^Head^-TW sub-module by comparing the conformations of Cenp-HIK-TW in the context of three states (i) the CCAN – Cenp-A^Nuc^ complex (37), (ii) the CCAN dimer (37), and (iii) the recently reported Cenp-HIK-TW complex (Ctf19c-Cnn1-Wip1) (57). Superimposing all three structures onto the structurally invariant Cenp-HIK^Body^ shows that Cenp-HIK^Head^-TW sub-module adopts a range of conformations facilitated by the flexible joint that connects Cenp-HIK^Body^ and Cenp-HIK^Head^-TW (Figure 4C and Supplementary Figure S5). In the CCAN–Cenp-A^Nuc^ complex, the conformation of Cenp-HIK^Head^-TW has swung out relative to that in the isolated Cenp-HIK-TW complex and apo dimeric CCAN, to create a straightened configuration for Cenp-HIK-TW. This conformational flexibility provides space for Cenp-A^Nuc^ in the CCAN–Cenp-A^Nuc^ complex.

### Model for (CCAN)_2_–Cenp-A^Nuc^

**Figure 6.**
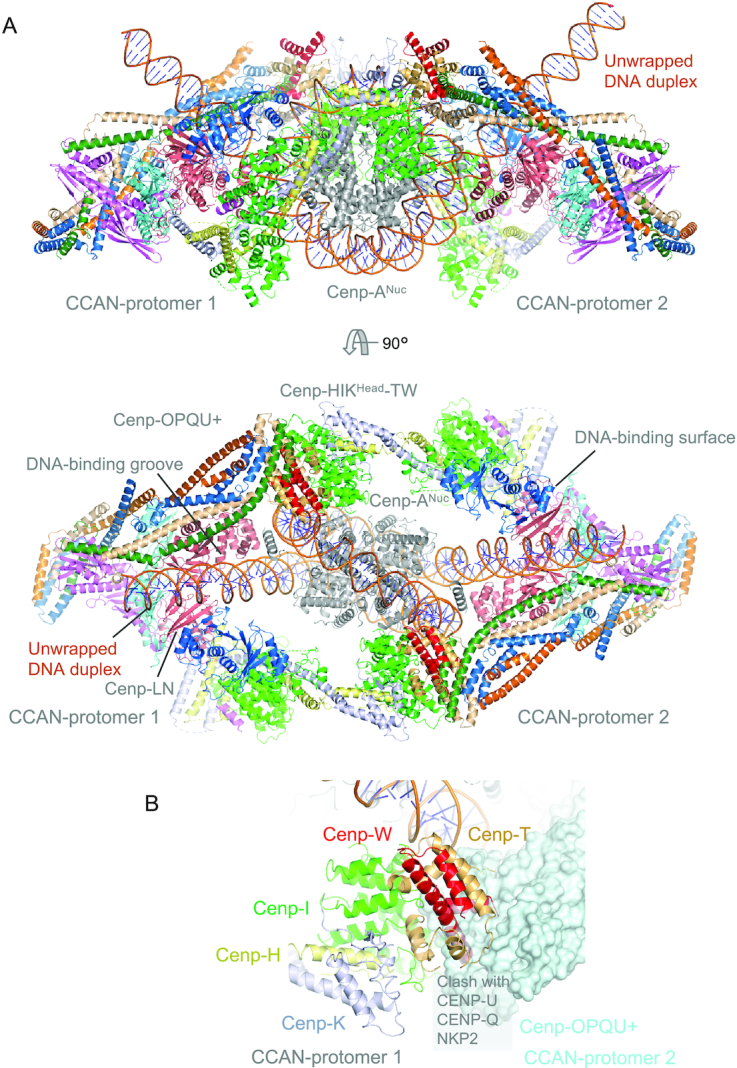
Model for the (CCAN)_2_ – Cenp-A^Nuc^ complex. (**A**) Two orthogonal views of the complex showing how Cenp-A^Nuc^ slots into the groove formed by the association of two ‘Y’-shaped CCAN protomers. The unwrapped DNA at the two Cenp-A^Nuc^ termini engage the DNA-binding groove of Cenp-LN at the centre of each CCAN protomer. (**B**) Close-up view of the interaction between Cenp-HIK^Head^-TW and Cenp-OPQU+ of the symmetry-related protomer with Cenp-OPQU+ shown in surface representation. There is a slight clash of Cenp-TW with Cenp-OPQU+.

Based on the dyad symmetry operator of Cenp-A^Nuc^, we generated a model for two CCAN protomers assembled onto a single Cenp-A^Nuc^ (Figure 6A). This model suggests how a Cenp-A nucleosome would be supported by the CCAN dimer with the unwrapped DNA ends of Cenp-A^Nuc^ interacting with the DNA-binding surface of CCAN. As reported previously, 2D classification of the cryo-EM dataset identified 2D projections consistent with such a model, and the molecular mass measurements determined using AUC and SEC-MALS (37). In the model there is some overlap of Cenp-TW with Cenp-U, Cenp-Q and Nkp2 of the symmetry-related CCAN protomer (Figure 6B). The conformational flexibility of Cenp-HIK^Head^-TW would be expected to allow for relief of this steric overlap.

## CONCLUDING REMARKS

The crystal structure of Cenp-HIK^Head^-TW reported here provides the missing structural data to complete the CCAN atomic model, and defines the interface between Cenp-HIK and Cenp-TW. Fitting the Cenp-HIK^Head^-TW atomic model to the cryo-EM map of CCAN – Cenp-A^Nuc^ indicates that the positively charged surface of both Cenp-I^Head^ and Cenp-T interact with the DNA gyre of Cenp-A^Nuc^. This agrees with previous data that Cenp-TW binds DNA (12). Although we think it unlikely that budding yeast Cenp-TWSX forms a nucleosome-like particle at a distinct locus from the *Cen* locus, our study leaves open the possibility that the budding yeast Cenp-SX orthologs interact with Cenp-TW in the context of chromatin. The model of CCAN–Cenp-A^Nuc^ with the fitted Cenp-HIK^Head^-TW provides a framework for understanding how Cenp-T links centromeric Cenp-A^Nuc^ to the outer kinetochore through its N-terminal Ndc80 complex-binding motif.

While this manuscript was in preparation, the cryo-EM structure of the *S. cerevisiae* Ctf3c-Cnn1-Wip1 (Cenp-HIK-TW) complex was deposited (PDB: 6WUC) (57). In this structure, the Cenp-HIK–Cenp-TW interface, involving the HFE of Cenp-T is well defined, with conclusions similar to ours. However, the Cenp-T^E346^-Cenp-I^R173^ salt-bridge interaction is not formed, and in addition, the HFDs of Cenp-T and Cenp-W, which were not well resolved in the cryo-EM maps, were modelled on the chicken Cenp-TW coordinates. For Cenp-W, α-helix H1, built in our structure as residues 1–15, was modelled as residues 15–25 in 6WUC.

## DATA AVAILABILITY

Protein coordinates and MTZ file have been deposited with RCSB, ID: 6YPC. The cryo-EM map of CCAN – Cenp-A^Nuc^ used to fit CCAN – Cenp-A^Nuc^ including the Cenp-HIK^Head^-TW sub-module was deposited with EMD, ID EMD-11626.

## Supplementary Material

gkaa772_Supplemental_File
