# Supplementary material for: Crystal structure of the Cenp-HIKHead-TW sub-module of the inner kinetochore CCAN complex
Source: Nucleic Acids Res. 2020 Sep 25;48(19):11172–84. doi: 10.1093/nar/gkaa772 (PMC7641736; doi:10.1093/nar/gkaa772)
Supplement: gkaa772_Supplemental_File [file gkaa772_supplemental_file.pdf]

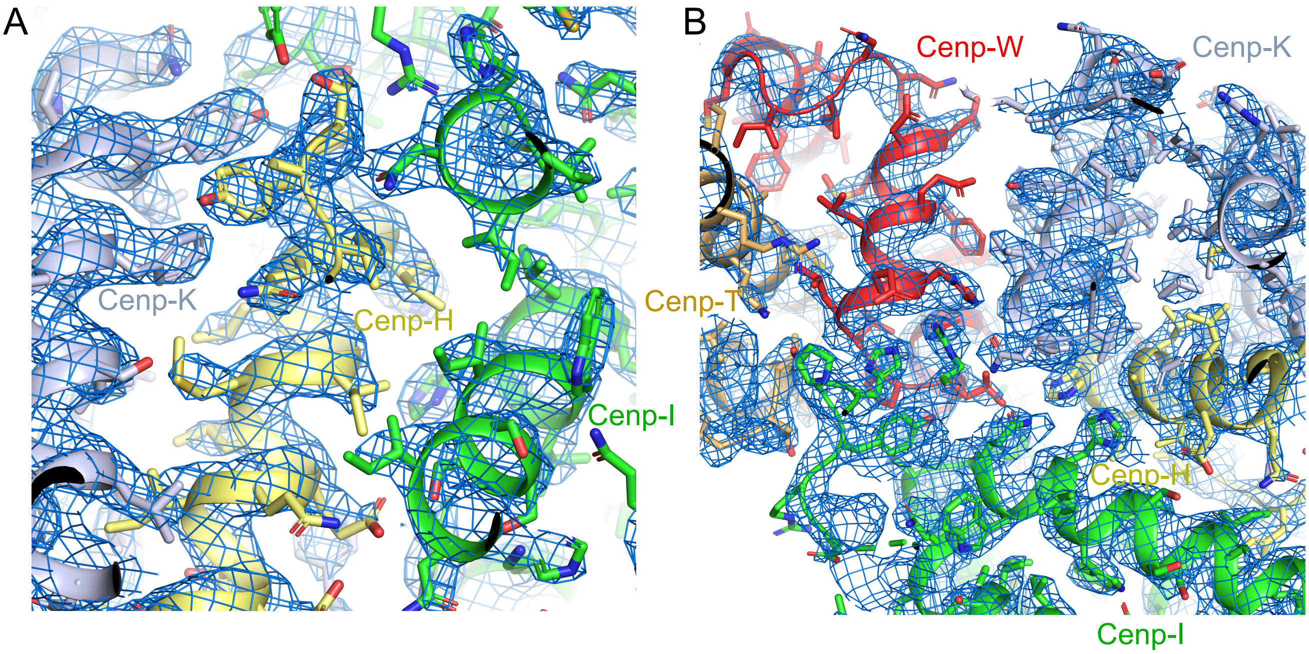

Supplementary Figure S1. Sections of the final 2Fo-Fc electron density map of the refined Cenp-HIK^{Head}-TW sub-module. (**A**) and (**B**) Two sections of electron density at the Cenp-HIK^{Head} – Cenp-TW interface. The model is coloured as in **Figure 1**.

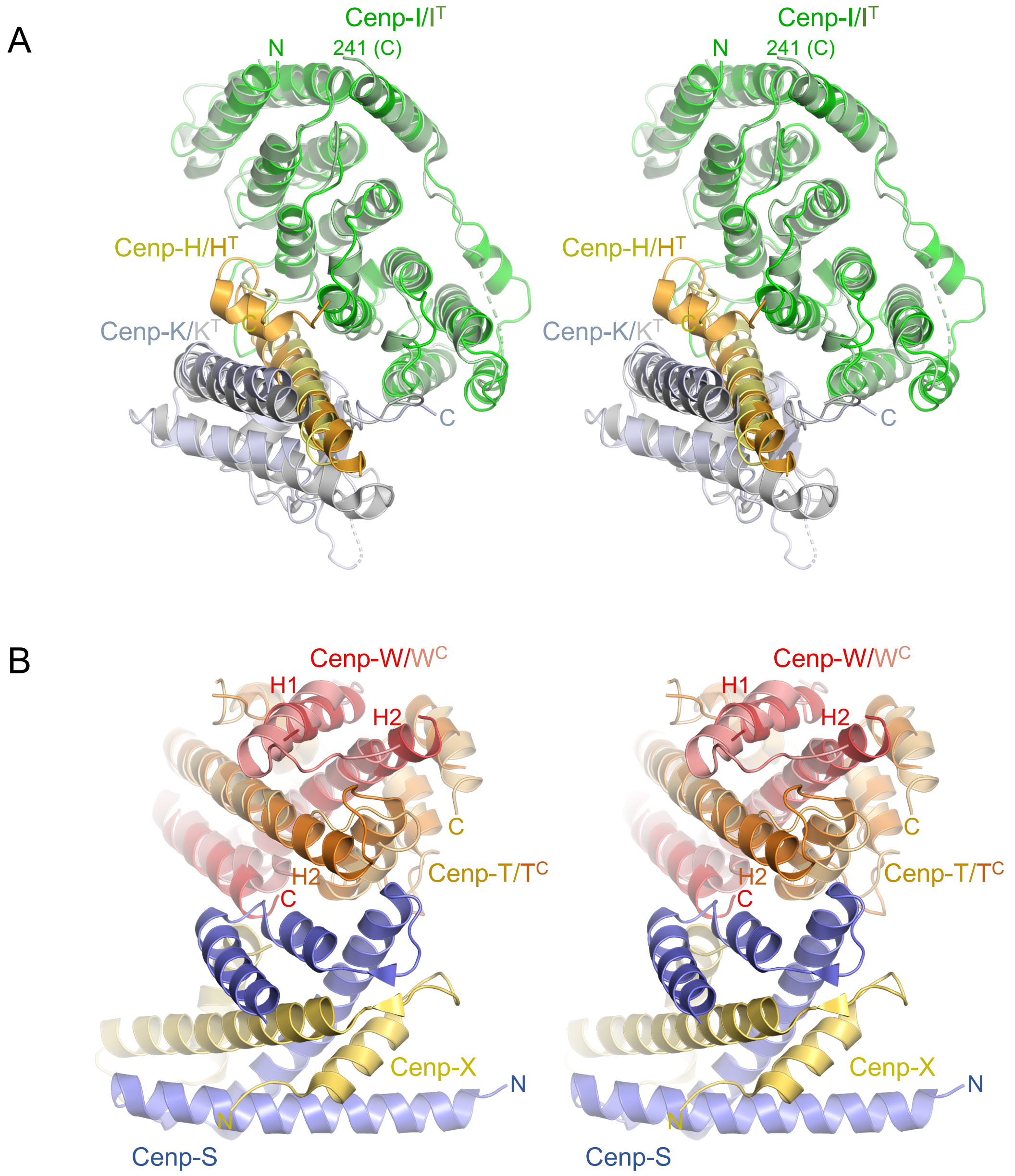

Supplementary Figure S2. Cenp-HIK^{Head} and Cenp-TW are structurally conserved. (**A**) Stereo-views showing a superimposition of *S. cerevisiae* Cenp-HIK^{Head} onto thermophilic yeast Cenp-HIK^{Head} [PDB: 5Z08] (38). Superscript 'T' denotes thermophilic yeast. (**B**) Stereo-views showing a superimposition of *S. cerevisiae* Cenp-TW onto chicken Cenp-TWSX, [PDB: 3VH5] (15). Superscript 'C' denotes chicken.

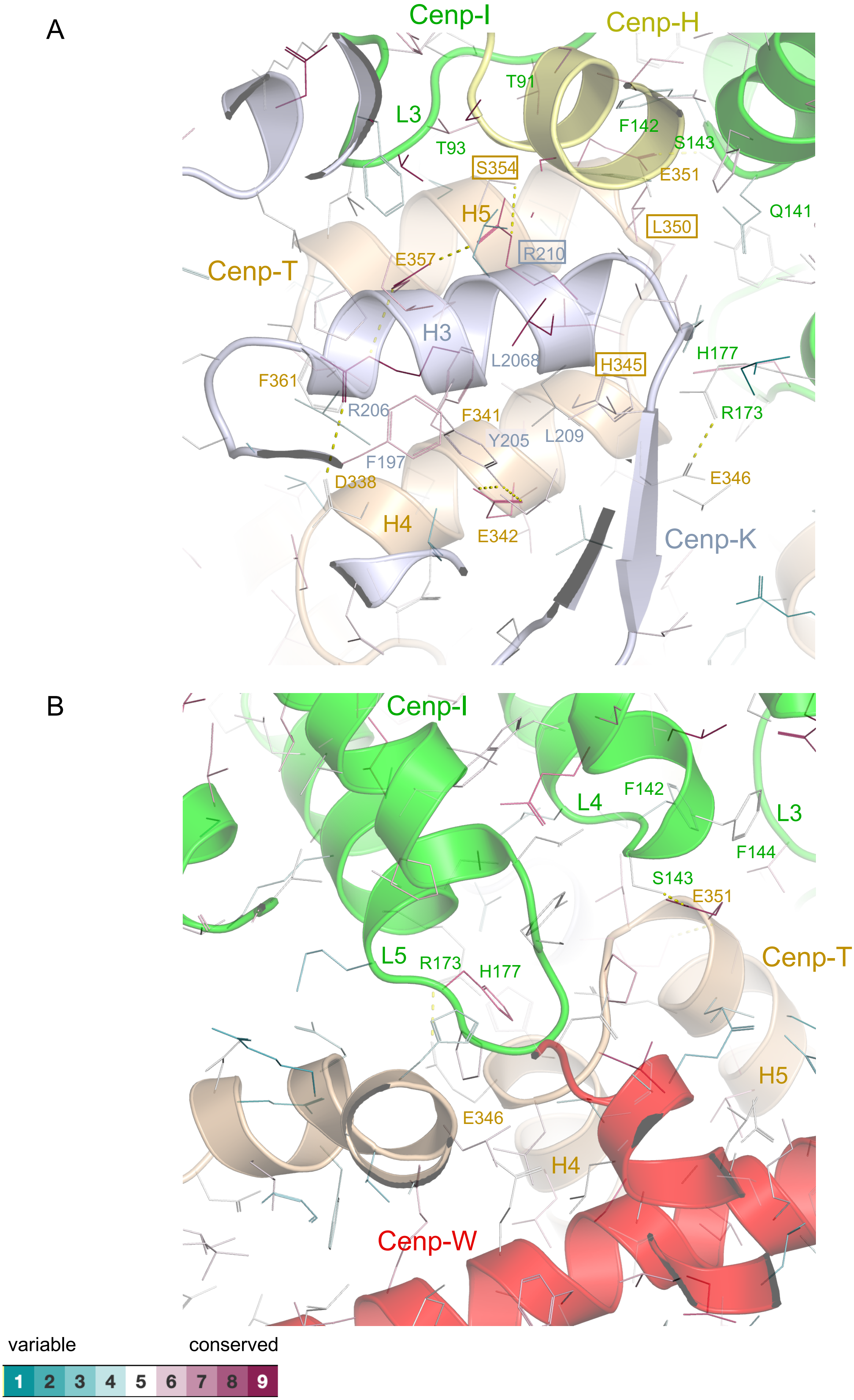

Supplementary Figure S3. The Cenp-HIK^{Head} – Cenp-TW interface is conserved. (**A**) Details of residues forming the interface 1. (**B**) Details of residues forming the interface 2. Conservation analysis was performed using Consurf (54,55).

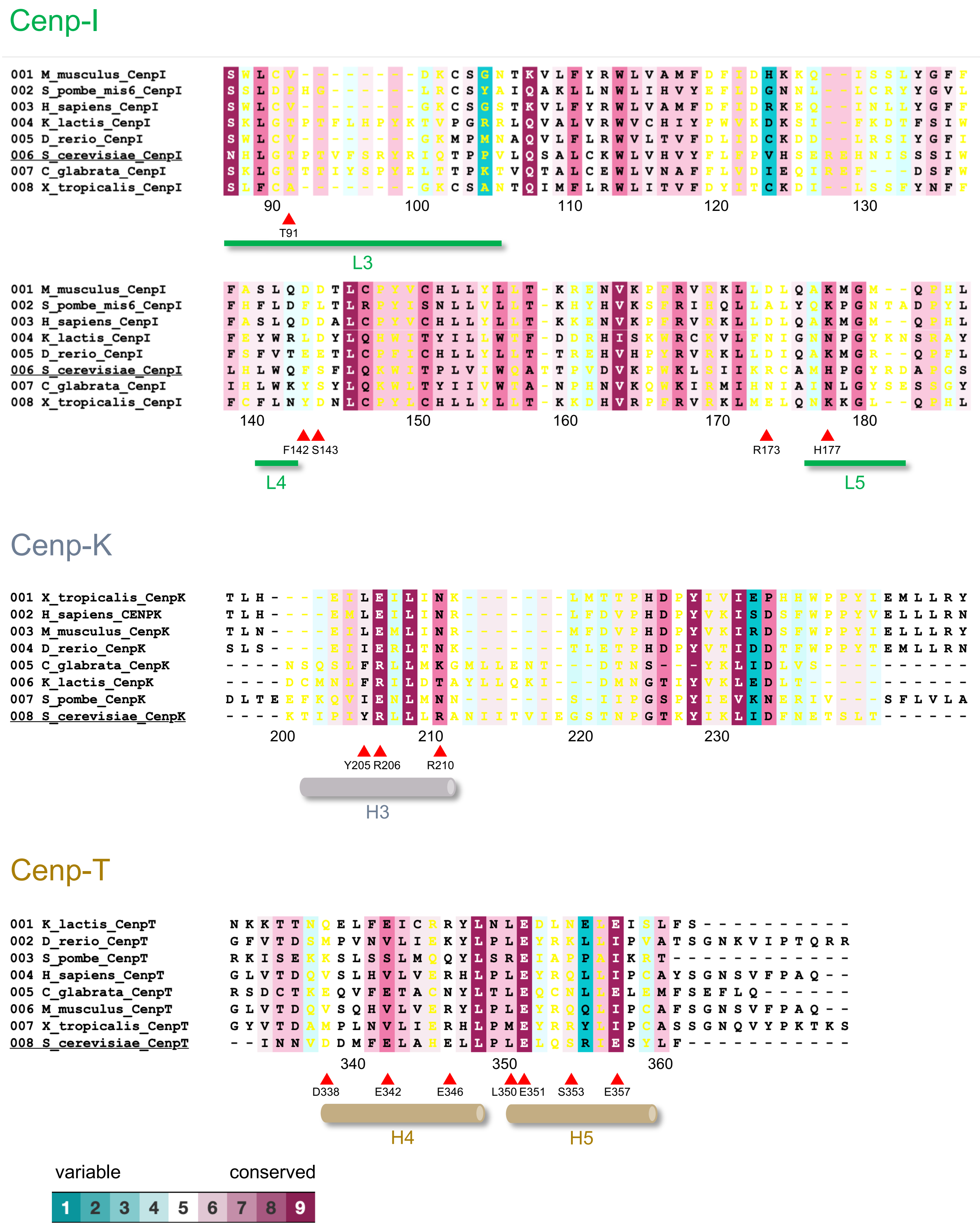

Supplementary Figure S4. Multiple sequence alignment of regions of Cenp-I, Cenp-T and Cenp-T forming the Cenp-HIK^{Head}-TW interface. Output from Consurf (54,55). Key residues are indicated with a red up arrow. Secondary structural elements are indicated.

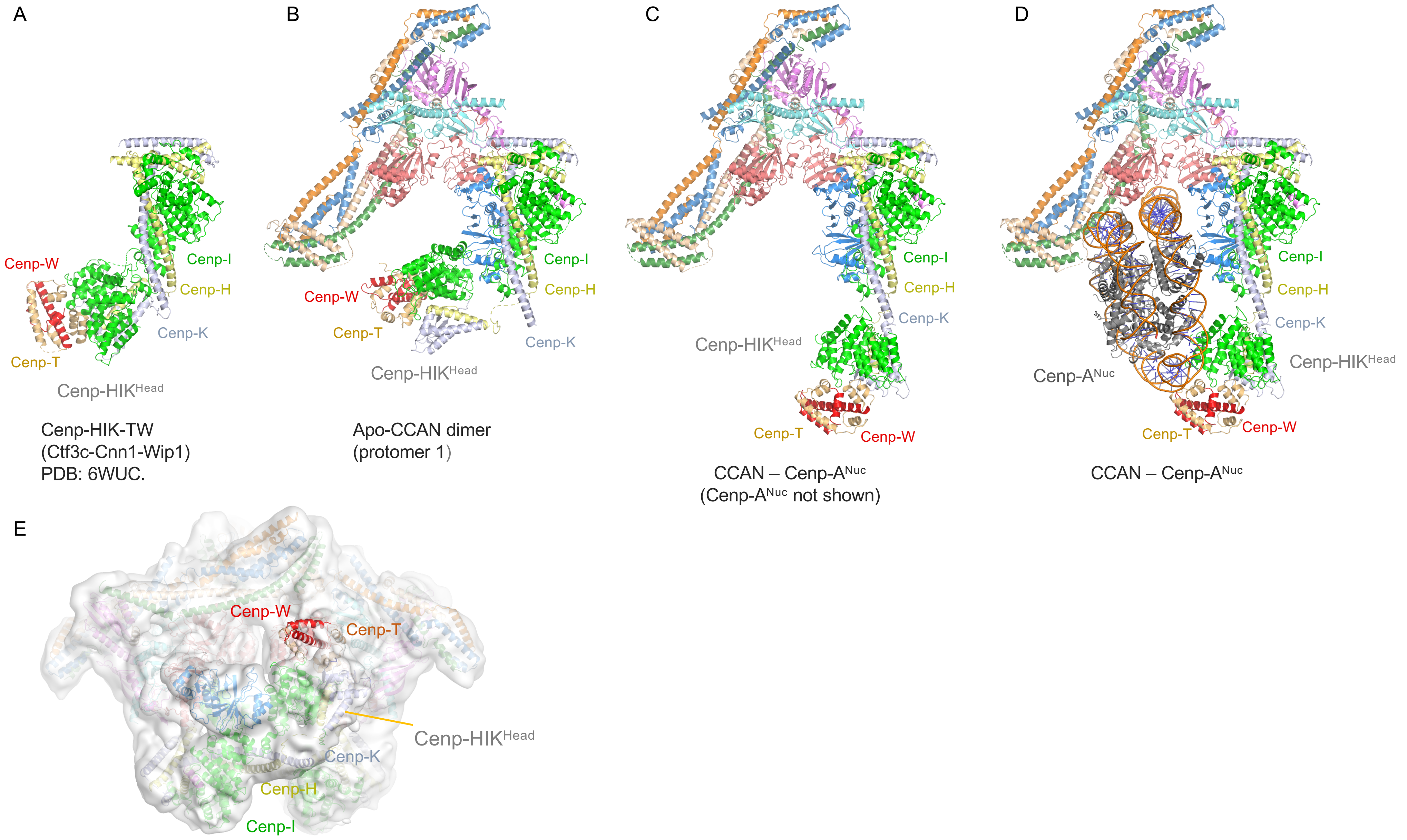

Supplementary Figure S5. Cenp-HIK^{Head}-TW is conformationally flexible. Structures of the Cenp-HIK^{Head}-TW sub-module in different contexts: (**A**) Cenp-HIK-TW (Ctf3c-Cnn1-Wip1) cryo-EM structure (PDB 6WUC) (57), (**B**) apo-CCAN dimer (protomer 1) (37), (**C**) CCAN – Cenp-A^{Nuc} cryo-EM structure (Cenp-A^{Nuc} not shown) (37), (**D**) CCAN – Cenp-A^{Nuc} cryo-EM structure. (**E**) The crystal structure of Cenp-HIK^{Head}-TW is fitted to the cryo-EM density map of CCAN dimer (37). Cryo-EM map shown as transparent surface. CCAN model is shown in cartoon representation.
